# Comparison of Antioxidant Status and Vitamin D Levels between Multiple Sclerosis Patients and Healthy Matched Subjects

**DOI:** 10.1155/2014/539854

**Published:** 2014-04-14

**Authors:** Ehsan Hejazi, Reza Amani, Naser SharafodinZadeh, Bahman Cheraghian

**Affiliations:** ^1^Department of Clinical Nutrition and Dietetics, Faculty of Nutrition Sciences and Food Technology, National Nutrition and Food Technology Research Institute, Tehran, Iran; ^2^Department of Nutrition, School of Para Medicine, Diabetes Research Center, Jundishapur University of Medical Sciences, Ahvaz 61357-15794, Iran; ^3^Department of Neurology, Golestan Hospital, Jundishapur University of Medical Sciences, Ahvaz, Iran; ^4^Department of Epidemiology, School of Public Health, Jundishapur University of Medical Sciences, Ahvaz, Iran

## Abstract

*Objective*. The aim of the present study was to compare the serum levels of total antioxidant status (TAS) and 25(OH) D3 and dietary intake of multiple sclerosis (MS) patients with those of normal subjects. *Method*. Thirty-seven MS patients (31 women) and the same number of healthy matched controls were compared for their serum levels and dietary intake of 25(OH) D3 and TAS. Sun exposure and the intake of antioxidants and vitamin D rich foods were estimated through face-to-face interview and food frequency questionnaire. *Results*. Dietary intake of antioxidants and vitamin D rich foods, vitamin C, vitamin A, and folate was not significantly different between the two groups. There were also no significant differences in the mean levels of 25(OH) D3 and TAS between the study groups. Both groups had low serum levels of 25(OH) D3 and total antioxidants. *Conclusion*. No significant differences were detected in serum levels and dietary intake of vitamin D and antioxidants between MS patients and healthy controls. All subjects had low antioxidant status and vitamin D levels.

## 1. Introduction

Multiple sclerosis (MS) is an autoimmune, inflammatory, and demyelinating disease which can cause significant disability among young adults [1, 2]. The etiology of MS has not been fully understood yet; however, it is believed that immunological mechanisms are the most important factors in initiation and progression of the disease [3]. It is generally accepted that vitamin D status is related to both innate and adaptive immune system [4]. There is considerable body of evidence, including prospective cohort studies, that shows that increased levels of sun exposure [5], greater dietary vitamin D intake [6], or higher levels of serum 25(OH) D3 are correlated with a lower risk of MS onset. Several studies have demonstrated the preventive effect of vitamin D on the onset or development of animal model of MS, experimental autoimmune encephalomyelitis (EAE) [7, 8]. It has been also proven that oxidative stress has an essential role in the inflammatory processes and in the pathogenesis of MS [9]. Oxidative and nitrosative stress may cause selective oligodendrocyte death, and thereby demyelination. The reactive species may also damage the myelin sheath, promoting its attack by macrophages [8]. It has been also reported that free radicals are necessary to phagocytize myelin by macrophage as well [10]. Furthermore, because of the vulnerability of the Central Nervous System (CNS) to the reactive oxygen species (ROS), decreased cellular antioxidant defense in CNS can increase the injury observed in MS [11]. Measurement of serum concentrations of different oxidant species can be time consuming and expensive; hence, total antioxidant status (TAS) is an effective estimate of the activity of blood antioxidants [12]. Studies on patients with MS with respect to serum 25(OH) D3 and 1, 25-(OH)2 D3 and TAS concentrations are rare. Therefore, in this case control study, we have evaluated blood concentrations of hydroxyl vitamin D and TAS and also dietary intakes of the main antioxidants and vitamin D sources in MS patients and age- and sex-matched healthy controls.

## 2. Materials and Method

Thirty-seven patients with relapsing-remitting multiple sclerosis (RRMS), who were referred to the Clinic of Neurology in Golestan Medical Center, Ahvaz Jondishapur University, by a Neurologist (NSZ), were enrolled in the present case-control study. Diagnosis was confirmed on the basis of MRI and clinical or laboratory-supported diagnosis of definite RRMS MS [13]. All patients were in remitting phase and their Expanded Disability Status Scale (EDSS) was low (EDSS < 4.5).

Thirty-seven sex- and age-matched controls were recruited from healthy blood donors in the same urban catchment area. All participants in this study were living for a long time in the city of Ahvaz.

Exclusion criteria for both groups were having hepatic or renal diseases, type 1 diabetes, heart disease, hypercortisolism, and pregnancy, and also intake of multivitamin supplements, alcohol consumption, and Smoking. Five patients were excluded from the study because of the difficulty in measurement of their vitamin D and TAS levels. After an overnight fasting, whole blood samples of patients were drawn. The serum samples were then stored at −20°C and protected from direct exposure to sunlight until further analysis. Serum 25 hydroxy vitamin D concentrations were measured using RIA method (Biosource Europe) [12, 14]. Vitamin D levels equal to or below 12.5 nmol/L were considered as severe deficiency. Concentrations between 12.5 nmol/L and 25 nmol/L were considered as moderate deficiency and levels between 25 nmol/L and 35 nmol/L were regarded as mild deficiency [14]. Plasma TAS was measured by Hitachi Analyzer with a Randox reagent set (England). The radical cation obtained has a relatively stable blue-green color, which is measured at 600 nm. Antioxidants contained in the serum sample suppress the formation of this color [15]. The subjects were asked to complete a sun light exposure questionnaire which its validity and reliability were confirmed [16]. The questionnaire was containing details about duration of exposure to sun light in the previous month (less than 30, between 30 to 60, between 60 to 120, and more than 120 minutes/day), sunscreen cream usage and clothing (exposure of hand and face or more areas). Information about dietary intake of vitamin D sources and dietary antioxidants was obtained using a semiquantitative food frequency questionnaire [9]. Daily intake of vitamin C, vitamin E, vitamin A, and folate was recorded from a 24-hour dietary recall questionnaire for 3 days (2 consecutive and one weekend days). Dietary data were analyzed using Nutritrack software (2004 version). Subjects' weights and heights were measured using a platform digital scale (Seca, Germany) and nonstretchable wall stadiometer, respectively. BMI was defined as weight divided by squared height (kg/m^2^). SPSS version 13 was used for statistical analysis and a *P* value < 0.05 was considered as statistically significant.

## 3. Result

Thirty-seven patients were recruited in each group. The age ranges of participants were between 18 to 52 years. Eighty-four percent of them were female. All of the participants have completed the requested questionnaires.

Patient demographic characteristics and mean levels of 25(OH) D3 and plasma TAS are presented in Table 1. There were no significant differences in basic variables such as age, sex, BMI, mean levels of 25(OH) D3, and TAS between the study groups. According to Table 2, there is no relationship between levels of 25(OH) D3 and sun exposure in MS patients. However, in healthy subjects, more sun exposure was related to higher levels of 25(OH) D3 (*P* < 0.05).  Table 3 shows that there is no significant association between fish and egg consumption and serum levels of 25(OH) D3. However, in healthy subjects with egg consumption more than 3 times per week, it means that serum vitamin D concentration was higher compared with those who consumed 1-2 eggs per week.  Table 4 shows that daily intake of vitamin C, vitamin E, vitamin A, and folate was not significantly different between the study groups. The intake of all antioxidant vitamins in both groups was well below the dietary reference intake (DRI). However, daily intake of vitamin C in control group was near to RDA.

## 4. Discussion

The aim of the present study was to compare the serum levels of 25(OH) D3 and TAS, dietary intake of the main antioxidants, and vitamin D sources in MS patients with those of healthy subjects. In the present study, we could not find any significant association between the duration of sunshine exposure and serum levels of 25(OH) D3 in the patients. There are only few studies which have compared serum levels of 25(OH) D3 between MS patients and healthy controls [17–20]. Some of them have reported that the serum levels of 25(OH) D3 in MS patients are lower in comparison with healthy controls [18], whereas in a study by Soilu-Hänninen et al., no significant differences were observed between serum levels of 25(OH) D3 in MS patients and healthy controls in winter [20]. Our findings are in line with Soilu-Hänninen et al., probably because of the similarity in the time of data gathering and sex of the subjects. In a case control study in Australia [21], it was reported that the patients with higher disability (EDSS* > 6) were more likely to have vitamin D insufficiency compared with controls (OR = 3.07 (1.37, 6.90)); however, there were no differences between the serum levels of 25(OH) D3 of patients with lower disability (EDSS > 3) and controls. Since the majority of patients (65%) in the present study had low disability (EDSS < 3), lack of the significant differences between the 2 groups is in agreement with the latter study. In a case control study by Shaygannejad, lower levels of serum 25(OH) D3 in MS patients compared with healthy controls were reported [17]; however, the severity of the disease was not considered in this study.

In the present study, severe vitamin D deficiency (25(OH) D3 < 12.5 nmol/L) was observed in about one-third of the participants (33% of patients versus 37% of controls), and most of the subjects (66% versus 73% of patients and controls, resp.) had moderate vitamin D deficiency. Several studies have reported high prevalence of vitamin D deficiency in MS patients [17, 18, 20, 22]. High prevalence of vitamin D deficiency in Iran and other countries in the Middle East was reported in several previous studies [16]. Hashemipour et al. have shown vitamin D deficiency in 81% of Tehranian healthy donors [16]. In Saudi Arabia and Lebanon, despite abundant sun exposure, high prevalence of vitamin D deficiency has been also reported [23]. Possible justifications for this finding could be pollution of air, hyper pigmentation of skin, religious clothing patterns, special dietary habits, and inadequate dietary intake of vitamin D [16]. Ahvaz in the south of Iran has a climate similar to these Arab countries and high prevalence of vitamin D deficiency despite its southern latitude can be justified in the same manner.

In the present study, sunshine exposure (between 10 AM and 2 PM) did not significantly affect serum 25(OH) D3 concentrations in patients, while the levels of 25(OH) D3 in healthy counterparts who had low sunshine exposure (<30 min) were lower (*P* < 0.005) than those of healthy subjects with a high exposure (>30 min/d). This discrepancy between sun exposure and concentrations of 25(OH) D3 was also reported in other studies [20, 24].

No significant differences were observed between the consumption of main dietary sources of vitamin D such as fish, egg, butter, and cream in MS patients and healthy controls. Some Previous studies which have compared dietary intake of vitamin D between MS patients and controls, have also reported similar findings. In a case control study, no significant differences in terms of dairy foods, fish, and eggs intake were observed between cases and controls [25]. In another investigation by Berr et al., intake of milk, dairy products, and fish was unrelated to the prevalence of MS [24]. Zhang et al. have also reported that intake of dairy products and fish was not significantly related to the risk of MS [26]. Because a large proportion of the body's vitamin D requirement is synthesized via sun exposure, these findings are rational. Our results also showed that there is no significant association between fish consumptions and serum 25(OH) D3 concentrations in both groups. In contrast, In a study on healthy peri- and postmenopausal Japanese women, it has been reported that 25(OH) D concentration of subjects who consumed fish frequently (≤4 times/wk) was 10.1 nmol/L higher than that of subjects with a moderate consumption of fish (1–3 times/wk) [27]. It is worth mentioning that fish consumption in Japan is much higher than that of Iran (70 kg/y versus 2.6 kg/y per capita, resp.), and Japan has one of the highest rates of fish consumption in the world.

Just few previous studies have compared the serum levels of TAS between MS patients and healthy subjects. According to the results of a study by Visconti et al. [28], there was no significant difference between the means of serial measures of serum antioxidant capacity in patients with first demyelinating episode (FDE) compared with a healthy population over a six-month period. On the other hand, Besler and Çomoğlu [29] have reported that MS patients had significantly lower plasma total antioxidant capacity compared with the control group. It seems necessary to note that, in the latter study, secondary progressive MS patients who first experienced exacerbations were recruited. In the present study, however, patients diagnosed as relapsing-remitting MS were included in the study during an attack-free phase. Variations in MS phases and disease severity may cause these discrepancies. Our results are in agreement with those of Koch et al.'s study [30] that found that serum total antiradical activity (ARA) and total antioxidant activity (AOA) were not significantly different between MS patients and healthy controls. However, our findings do not support those of Acar et al.'s [12] study that reported that MS patients had lower concentrations of total oxidative status (TOS) and TAS compared with healthy controls. These conflicting results are also likely to be related to different clinical conditions and/or different dietary habits of the patients, interactions of pharmacotherapies, or methodological issues [31, 32].

In a study by Besler et al., antioxidant vitamins' levels (alpha tocopherol, beta-carotene, retinol, and ascorbic acid) were decreased in serum of MS patients during an attack, and this was defined to be dependent on the increased oxidative burden as reflected by lipid peroxidation products [32]. In another study, MS patients had significantly lower serum uric acid levels in comparison with healthy donors. Also the authors found that MS patients with relapse had significantly lower serum uric acid levels compared with MS patients in remission phase [31]. It is noteworthy that the total antioxidant defense status of a cell determines the susceptibility of cells to oxidative stress instead of measuring the concentration of individual antioxidants (e.g., uric acid, antioxidant vitamins, bilirubin, and glutathione) or antioxidant enzymes (e.g., glutathione peroxidase and superoxide dismutase) [30]. Our results showed that there was no significant difference in consumption of dietary antioxidant sources between the 2 groups. However, the intake of all antioxidant vitamins in both groups was well below the dietary reference intake (DRI). The results of some other studies were also in accordance with our findings [10, 24, 25]. For example, in a large cohort study, Zhang et al. [10] found no associations between intake of fruits and vegetables and risk of MS.

One potential source of bias in the present and other retrospective studies could be due to this fact that patients may change their dietary habits because of clinical symptoms of MS before they were diagnosed with MS. Also the small sample size of the present study can be considered as a limitation.

In conclusion, we could not detect any differences between vitamin D and TAS between MS and healthy matched subjects. Dietary intake of vitamin D and antioxidant vitamins also showed no significant differences between the 2 groups. According to the Cochrane review of Farinotti et al. [33], evidence on the possible benefits and risks of vitamin supplementation and antioxidant supplements in MS is lacking. At the moment, more researches are required to assess the effectiveness of dietary interventions in MS.

## Figures and Tables

**Table 1:** Basic characteristics, 25(OH) D3 and TAS1 serum levels of study groups*.

Variables	Patients (*n* = 37)	Controls (*n* = 37)	*P*
Age (yr)	32 ± 0.8	32.2 ± 7.4	0.9
Sex (%F)	84	84	1
Height (cm)	160.8 ± 8	163.1 ± 10.3	0.3
Weight (Kg)	64.2 ± 10.6	66.5 ± 3.1	0.4
BMI (Kg/m²)	24.9 ± 3.9	24.9 ± 4.1	1
25(OH) D3 (nmol/L)	20.67 ± 16.3	15.8 ± 8.7	0.1
TAS (*μ*mol/L)	1 ± 0.1	1.03 ± 0.1	0.2

*Values are mean ± SD; TAS: total antioxidant status.

**Table 2:** Comparison of serum vitamin D concentrations based on different sun exposure levels.

Sun exposure (minutes/day)		MS patients		Controls	
		25(OH) D serum concentrations (nmol/L)	Number	25(OH) D serum concentrations (nmol/L)	Number
Less than 30		18.4 ± 16	20	**12.6** ± **5.9**	**22**
Between 30 and 60		24.2 ± 29.2	5	**21** ± **7.7** ^a^	**10**
Between 60 and 120		26.6 ± 13.2	10	**43.6** ± **14.1** ^a,b^	**5**
More than 120		23.1 ± 7.2	2	—	***0***

^a^Significant differences between those of <30 minutes/day of sun exposure (ANOVA, *P* < 0.05).

^b^Significant differences between those between 30 and 60 minutes/day of sun exposure (ANOVA, *P* < 0.05).

**Table 3:** Comparison of serum vitamin D levels based on frequency of dietary vitamin D intakes.

Foods	Frequency of intake (Per week)	MS patients		Controls	
		25(OH) D serum concentrations (nmol/L)	Number	25(OH) D serum concentrations (nmol/L)	Number
Fish	≥3	23.46 ± 26.65	6	19.5	1
	1-2	19.80 ± 12.44	20	16.92 ± 9.28	25
	rarely	22.32 ± 17.46	11	12.34 ± 7.34	11

Eggs	≥3	18.89 ± 14.87	11	19.2 ± 10.99*	14
	1-2	20.11 ± 14.97	16	12.7 ± 5.51	18
	rarely	23.65 ± 20.59	10	16.1 ± 9.2	5

*Significant differences between those with 1-2 per week intake (ANOVA, *P* < 0.05).

**Table 4:** Daily intake of antioxidant vitamins in study groups obtained from 24 h recall questionnaire*.

Variables	Patients (*n* = 37)	Controls (*n* = 37)	*P*
Vitamin A (*μ*g/d)	429.4 ± 192	439.7 ± 181	0.9
Vitamin E (mg/d)	6.9 ± 1	5.57 ± 1.7	0.3
Vitamin C (mg/d)	53.7 ± 31.9	70.8 ± 27.4	0.1
Folate (*μ*g/d)	287.7 ± 91.8	265.5 ± 76.1	0.8

*Values are mean ± SD.

Note: intake of all antioxidant vitamins was below the DRI recommendations (less than 66% DRI).
